# A GM1b/asialo‐GM1 oligosaccharide‐binding R‐type lectin from purplish bifurcate mussels *Mytilisepta virgata* and its effect on MAP kinases

**DOI:** 10.1111/febs.15154

**Published:** 2019-12-24

**Authors:** Yuki Fujii, Marco Gerdol, Sarkar M. A. Kawsar, Imtiaj Hasan, Francesca Spazzali, Tatsusada Yoshida, Yukiko Ogawa, Sultana Rajia, Kenichi Kamata, Yasuhiro Koide, Shigeki Sugawara, Masahiro Hosono, Jeremy R. H. Tame, Hideaki Fujita, Alberto Pallavicini, Yasuhiro Ozeki

**Affiliations:** ^1^ Graduate School of Pharmaceutical Sciences Nagasaki International University Sasebo Japan; ^2^ Department of Life Sciences University of Trieste Italy; ^3^ Department of Chemistry Faculty of Science University of Chittagong Bangladesh; ^4^ School of Sciences Yokohama City University Japan; ^5^ Department of Biochemistry and Molecular Biology Faculty of Science University of Rajshahi Bangladesh; ^6^ Department of Pharmacy Varendra University Rajshahi Bangladesh; ^7^ Graduate School of Medical Life Science Yokohama City University Japan; ^8^ Institute of Molecular Biomembrane and Glycobiology Tohoku Medical and Pharmaceutical University Sendai Japan; ^9^ Department of Biology and Evolution of Marine Organisms Stazione Zoologica Anton Dohrn Napoli Italy

**Keywords:** bivalves, ganglioside, *Mytilisepta virgata*, purplish bifurcate mussels, R‐type lectin

## Abstract

A 15‐kDa lectin, termed SeviL, was isolated from *Mytilisepta virgata* (purplish bifurcate mussel). SeviL forms a noncovalent dimer that binds strongly to ganglio‐series GM1b oligosaccharide (Neu5Acɑ2‐3Galβ1‐3GalNAcβ1‐4Galβ1‐4Glc) and its precursor, asialo‐GM1 (Galβ1‐3GalNAcβ1‐4Galβ1‐4Glc). SeviL also interacts weakly with the glycan moiety of SSEA‐4 hexaose (Neu5Acα2‐3Galβ1‐3GalNAcβ1‐3Galα1‐4Galβ1‐4Glc). A partial protein sequence of the lectin was determined by mass spectrometry, and the complete sequence was identified from transcriptomic analysis. SeviL, consisting of 129 amino acids, was classified as an R(icin B)‐type lectin, based on the presence of the QxW motif characteristic of this fold. SeviL mRNA is highly expressed in gills and, in particular, mantle rim tissues. Orthologue sequences were identified in other species of the family Mytilidae, including *Mytilus galloprovincialis*, from which lectin MytiLec‐1 was isolated and characterized in our previous studies. Thus, mytilid species contain lectins belonging to at least two distinct families (R‐type lectins and mytilectins) that have a common β‐trefoil fold structure but differing glycan‐binding specificities. SeviL displayed notable cytotoxic (apoptotic) effects against various cultured cell lines (human breast, ovarian, and colonic cancer; dog kidney) that possess asialo‐GM1 oligosaccharide at the cell surface. This cytotoxic effect was inhibited by the presence of anti‐asialo‐GM1 oligosaccharide antibodies. With HeLa ovarian cancer cells, SeviL showed dose‐ and time‐dependent activation of kinase MKK3/6, p38 MAPK, and caspase‐3/9. The transduction pathways activated by SeviL via the glycosphingolipid oligosaccharide were triggered apoptosis.

**Database:**

Nucleotide sequence data have been deposited in the GenBank database under accession numbers MK434191, MK434192, MK434193, MK434194, MK434195, MK434196, MK434197, MK434198, MK434199, MK434200, and MK434201.

## Graphical abstract

A 15‐kDa lectin (SeviL) was isolated from the purplish bifurcate mussel, *Mytilisepta virgata*. SeviL binds to gangliosides and was classified as an R‐type lectin. mRNA of SeviL was expressed in gills, and this orthologue was identified in another genus *Mytilus galloprovincialis* from which express mytilectins. Thus, the family Mytilidae has lectins belonging to two‐distinct families sharing β‐trefoil fold but differing glycan‐binding. SeviL displayed cytotoxicity against cultured cells. The transduction pathways activated by this lectin via glycosphingolipids triggered apoptosis.

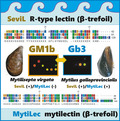

**Abbreviations**
Gb3: globotriaosylceramide
GM1: monosialotetrahexosylganglioside
MAPK: mitogen‐activated protein kinase
MKK: MAPK kinase
MytiLec‐1: *Mytilus galloprovincialis* ɑ‐Gal‐binding lectin
SeviL: *Mytilisepta virgata* R‐type lectin
SSEA‐4: stage‐specific embryonal antigen 4

## Introduction

Many marine invertebrates possess lectins (glycan‐binding proteins) with various glycan‐binding properties 1, 2, 3. In the differentiation of phylogeny, lectin‐mediated interactions between glycans and proteins were adapted into various kinds of key pathways involved in a variety of fundamental biological processes, including embryonic development, immune responses, and cell growth regulation 4, 5, 6. During this functional diversification, marine invertebrates developed an unusually large number of lectins, many having convergent structures that facilitate binding to specific glycan structures exposed on the surface of target cells. This combination of functional divergence and structural convergence has resulted in many unique sequences and unusual glycan‐binding specificities among lectins isolated from marine invertebrates 7, 8, 9, 10, 11.

We described in 2012 a novel lectin (termed ‘MytiLec‐1’), isolated from the Mediterranean mussel *Mytilus galloprovincialis* (family Mytilidae), that had a unique primary structure 12. MytiLec‐1 has a β‐trefoil fold 13, a 3‐D structure typically found in R‐type lectins, including ricin B‐chain 14. However, MytiLec‐1 bound specifically to the α‐galactoside globotriose (Galα1‐4Galβ1‐4Glc) 12, 13, whereas many other R‐type lectins bind to β‐galactosides such as N‐acetyllactosamine. MytiLec‐1 induced apoptosis in Gb3‐expressing human Burkitt’s lymphoma cells. Following the identification of similar lectins in various other mussel species 15, 16, 17, we referred collectively to such lectins as members of the ‘mytilectin family’ 18. The taxonomic distribution of mytilectins known to date is limited to the protostome clade *Lophotrochozoa* and discontinuous; members of this family have been identified only in the subclass *Pteriomorphia* (phylum Mollusca) and the order *Lingulida* (phylum Brachiopoda) 19.

Progress in ‘omics’ studies of mussels and other bivalve mollusks during the past decade has greatly enhanced our understanding of their genetics and molecular biology, leading to major advances in basic and applied scientific research. Mussels are a traditional seafood consumed heavily in Europe and increasingly in other parts of the world and are widely used as ‘sentinel’ organisms for biomonitoring 20. Molecular studies have revealed the essential role of lectins as pattern recognition receptors (PRRs) for microbe‐associated molecular patterns (MAMPs) in the innate immune systems of mussels 21, 22. A more complete understanding of these lectins will therefore provide a useful basis for improved mussel breeding practices and prevention of infections. Physiological processes and the immune system in mussels are strongly correlated with exposure to biotic and abiotic stress factors 23, 24. Numerous mussel immune system molecules including lectins were recently shown to be functionally modulated by pathogen exposure and ocean acidification 25, 26, so that bivalve lectins are suggested to be important molecules which respond to the marine environment. The large and highly diverse lectin repertoire of mytilids 27, 28, which probably includes several components yet to be identified, will facilitate effective new approaches for monitoring health status of mussel species, associated organisms, and their marine environments.

Since no R‐type lectin had been biochemically purified from the family Mytilidae, mytilectins were considered for some time to be the only β‐trefoil lectins present in mytilids, and it was speculated that their natural function related to the innate immune response 12, 15, 16, 17. However, the isolation of a novel lectin from the purplish bifurcate mussel (*Mytilisepta virgata*) in this study suggests the possibility of greater diversification among lectins in this family. Furthermore, the transcriptome of this species revealed a lack of mRNAs encoding mytilectins and instead revealed the expression of multiple distinct mRNAs encoding proteins characterized by the presence of a ricin B‐chain domain, typical of R‐type lectins 29. SeviL found from *M. virgata* shows characteristics of sugar chain binding and cell toxicity unlike any lectin reported to date. SeviL activated intracellular signaling pathways that resulted in cell death of mammalian carcinoma cells expressing asialo‐GM1, whereas MytiLec‐1 binds to Gb3 glycan. This is the first report that two different β‐trefoil lectin families (each with its own glycan‐binding specificity) coexist in the same animal species.

## Results

### Purification of lectin (SeviL) from *M. virgata*

**Figure 1:**
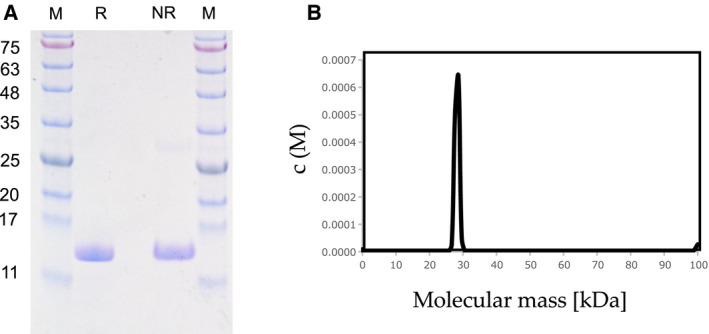
Purification of *Mytilisepta virgata* lectin, SeviL. (A) SDS/PAGE pattern under reducing (R) and nonreducing (NR) conditions. Numbers on the left indicate molecular masses (kDa) of marker proteins (M). (B) The molecular weight of the native protein (30 kDa) obtained from distribution of sedimentation coefficient by sedimentation velocity AUC. It indicates the presence of dimers in solution, with negligible amounts of monomer. Concentration c(M) was measured in absorption units (A_280_).

Supernatant ‘Sup 1‘(see Materials and methods ‘Lectin purification’) from homogenized *M. virgata* tissues displayed hemagglutination activity despite the absence of MytiLec‐1 from this species. Repeated homogenization of precipitates yielded supernatants with successively reduced activity (data not shown). The precipitates were homogenized again with 50 mm lactose to obtain supernatants. The hemagglutinating activity was recovered by dialyzing the supernatant ‘Sup 2’ (see Materials and methods ‘Lectin purification’). Sup 1 and 2 were applied to a lactosyl‐agarose column, and the new lectin could be eluted with TBS containing 50 mm lactose (Fig. 1A). The lectin was characterized as a single polypeptide with molecular mass 15 kDa by SDS/PAGE under both reducing and nonreducing conditions (Fig. 1A) and was termed ‘SeviL’. Purification from 400 g fresh tissue yielded 6.5 mg SeviL (Table 1). Hemagglutination activity of SeviL was required to the addition of calcium chloride (Fig. S1A), indicating that the activity was dependent on divalent cations such as Ca^2+^. Analytical ultracentrifugation revealed that SeviL was a tightly but noncovalently bound dimer (Fig. 1B).

**Table 1:** Purification of SeviL from *Mytilisepta virgata*.

Fraction	Titer (HU)	Volume (mL)	Total activity^a^	Protein conc. (mg·ml^−1^)	Protein amount (mg)	Specific activity^b^	Purification ratio (fold)^c^	Recovery of activity (%)^d^
Crude extract obtained by TBS	128	500	64 000	6.5	3525	0.04	1	100
Purified lectin	512	10	5120	0.27	2.7	190	4750	8
Crude extract obtained by lactose in TBS	512	100	51 200	2.1	2100	0.24	1	100
Purified lectin	4096	10	40 960	0.38	3.8	1024	4491	80

a Total activity is shown by Titer × volume

b Specific activity was shown by titer per mg of protein

c Purification ratio was shown by comparing the value of specific activity on the crude extract vs. purified lectin

d Recovery of activity was revealed by comparing the value of total activity on the crude extract vs. purified lectin.

### Sugar‐binding specificity of SeviL

Sugar‐binding specificity of SeviL is summarized in Table 2. Hemagglutination activity was weakly inhibited by addition of monosaccharides such as D‐Gal (25 mm), D‐GalNAc (25 mm), and D‐Fuc (25 mm) and of disaccharides such as melibiose (25 mm) and lactose (25 mm). These findings suggest that the chirality of the C3 and C4 carbons in galactose is essential for protein–glycan interaction. Hemagglutination activity was inhibited by administration of bovine submaxillary mucin (0.125 mg·mL^−1^), but not porcine stomach mucin or fetuin, even at concentrations > 1 mg·mL^−1^ (Table 2). These findings suggested that SeviL does not bind to porcine stomach mucin or fetuin, possibly because these glycoproteins have clusters of GlcNAc or sialyllactosamine at the reducing end 30.

**Table 2:** Saccharide and glycoprotein specificity of SeviL^a^.

Saccharides	Minimum inhibitory concentration (mm)
D‐GalNAc	25
D‐GlcNAc	N.I.^b^
D‐Gal	25
D‐Glc	> 50
D‐Man	> 50
D‐Fuc	25
Lactose	25
Melibiose	25
Sucrose	N.I.^b^

Glycoproteins	Minimum inhibitory concentration (mg·mL^−1^)
Bovine submaxillary mucin	0.125
Fetuin	N.I.^c^
Porcine stomach mucin	N.I.^c^

a Titer of SeviL was previously diluted to 16

b Inhibition was not occurred even at 200 mm

c Fetuin and bovine submaxillary mucin did not inhibit even at 2 mg·mL^−1^.

### Deduced primary structure of SeviL

**Figure 2:**
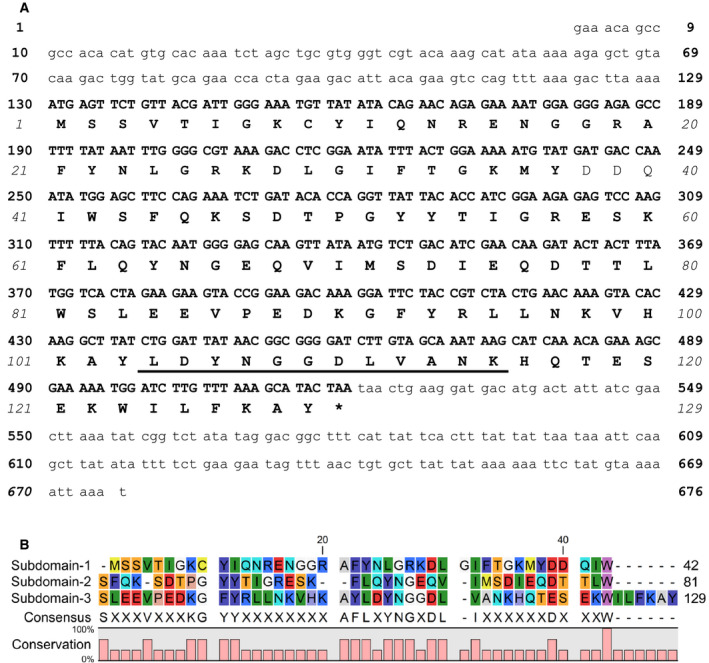
cDNA sequence and deduced amino acid sequence of SeviL. (A) The asterisk indicates the stop codon. The peptide fragment obtained from mass spectrometric analysis is underlined. (B) Amino acid sequence alignment of the internal tandem‐repeat subdomains of SeviL. The consensus at bottom summarizes the residues shared by the three domains. The sequence alignment within the polypeptide is analyzed by using MUSCLE program 31.

The cDNA sequence of SeviL was identified using a combination of *de novo* peptide sequencing and our earlier transcriptomics results 29. The peptide sequence obtained from trypsin digestion of SeviL (*m*/*z* 685.81 (MH_2_)^2+^) was LDYN(M/T/S/C) GDLVANK (Fig. S1B), which we compared with the mRNA sequences of five *M. virgata* tissues determined previously. A single sequence match was found with one protein product including the sequence ^104^LDYNGGDLVANK^115^ (Fig. 2A). The complete 129 amino acid residue sequence was classified as an R‐type lectin by the Pfam protein database (http://pfam.xfam.org/) but is unrelated to MytiLec‐1. At least two distinct lectin types (R‐type lectins and mytilectins) having the β‐trefoil fold structure are evidently present in the family Mytilidae.

### Tandem‐repeat structure of SeviL

SeviL has a triple tandem‐repeat structure with three subdomains, each consisting of ~ 40 amino acids, with 13–21% sequence similarity, consistent with a β‐trefoil fold (Fig. 2B). A QxW motif is conserved in each subdomain of R‐type lectins, and SeviL shows a similar pattern at residues 40–42 (QxW), 79–81 (TxW), and 121–123 (ExW). The SeviL sequence included only one Cys (C) residue, which does not form a disulfide bond (Fig. 1A).

A second sequence, highly homologous to SeviL and named SeviL‐2, was identified in the *M. virgata* transcriptome. The two sequences only differ at 10 out of 129 amino acid residues (Fig. S1C). Due to the high heterozygosity of mussels, SeviL and SeviL‐2 may either represent allelic variants of the same locus or the product of distinct orthologue genes, but the absence of a reference genome for this species hampered an in‐depth investigation. All the experiments carried out and reported in this paper refer to SeviL‐1, as the analysis of RNA‐sequencing data 29 revealed that this variant was expressed > 4‐fold higher than SeviL‐2 in all tissues.

### Comparison of SeviL orthologues among mytilid species

**Figure 3:**
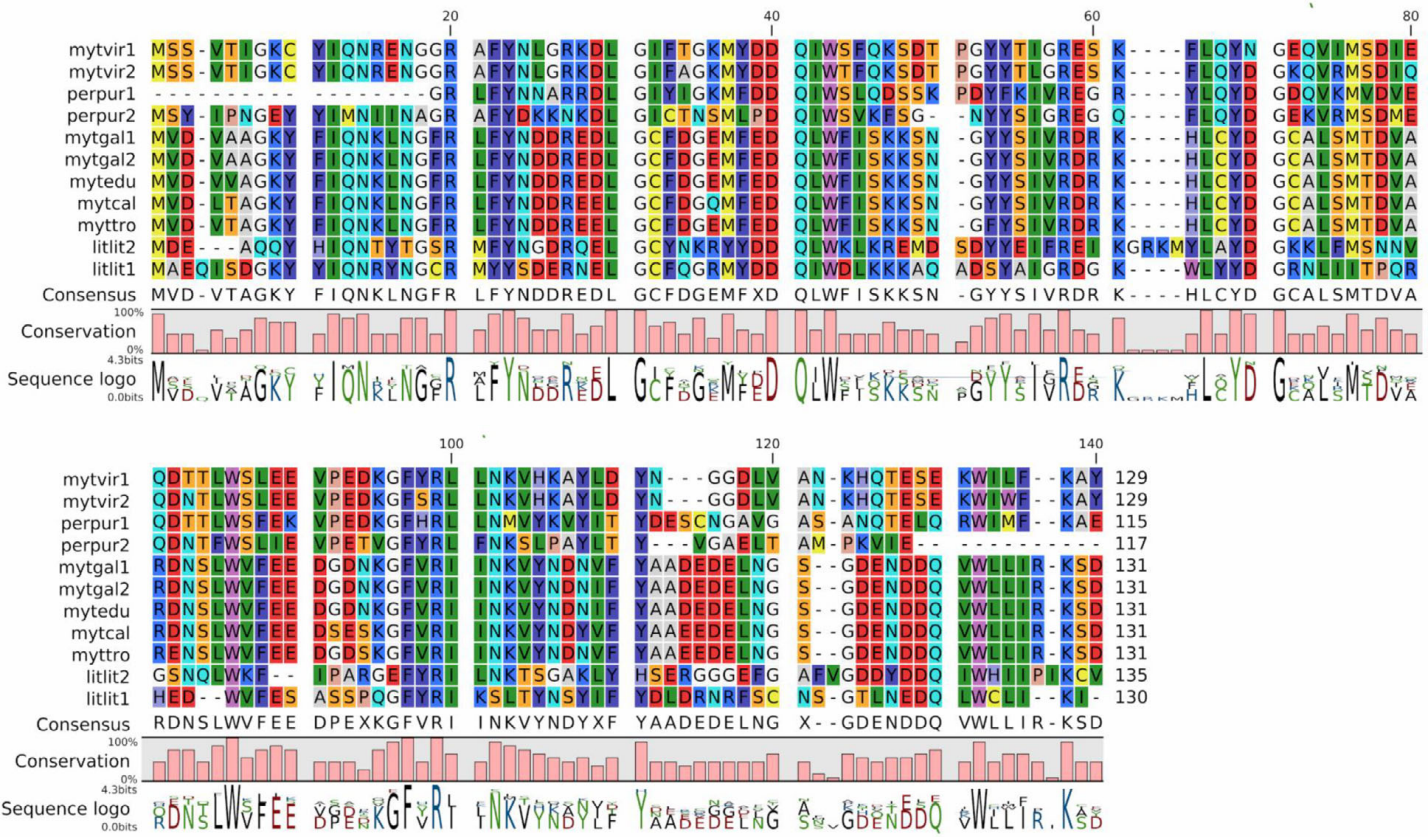
Multiple sequence alignment of SeviL orthologues in various mytilid species, detected in publicly available genomic or transcriptomic resources. mytvir1 (GenBank accession number MK434191) and mytvir2 (MK434192): *Mytilisepta virgata*; mytgal1 (MK434193) and mtgel2 (MK434194): *Mytilus galloprovincialis*; litlit1 (MK434195) and litlit2 (MK434196): *Lithophaga lithophaga*; mytedu (MK434197): *Mytilus edulis*; mytcal (MK434198): *Mytilus californianus*; myttro (MK434199): *Mytilus trossulus*; perpur1 (MK434200) and perpur2 (MK434201): *Perumytilus purpuratus*. Names with numbers (*e.g.*, mytvir1, mytvir2) indicate lectin variants from the same organism. Note that the sequence perpur1 and perpur2 display incomplete N‐terminal and C‐terminal regions, respectively. The sequence alignment within the polypeptide is analyzed by using MUSCLE program 31.

One or more SeviL orthologues are found in several other members of the family Mytilidae, including *M. galloprovincialis*, *M. edulis*, *M. californianus*, and *M. trossulus*, *P. purpuratus* and *L. lithophaga*, with a level of interspecies sequence conservation ranging from 25% to 99%, depending on the species considered (Fig. S2A). Like the aforementioned case of *M. virgata*, some species (i.e., *P. purpuratus*, *M. galloprovincialis,* and *L. lithophaga*) display two sequence variants, which were characterized more in detail in the Mediterranean mussel genome 32, revealing a two exons/one intron gene organization, with the ORF entirely contained within exon 2 (Fig. S2B). The three Trp residues of SeviL (residues 43, 81, 123; Fig. 3) are notably conserved in all of Mytilidae SeviL‐like proteins.

**Figure 4:**
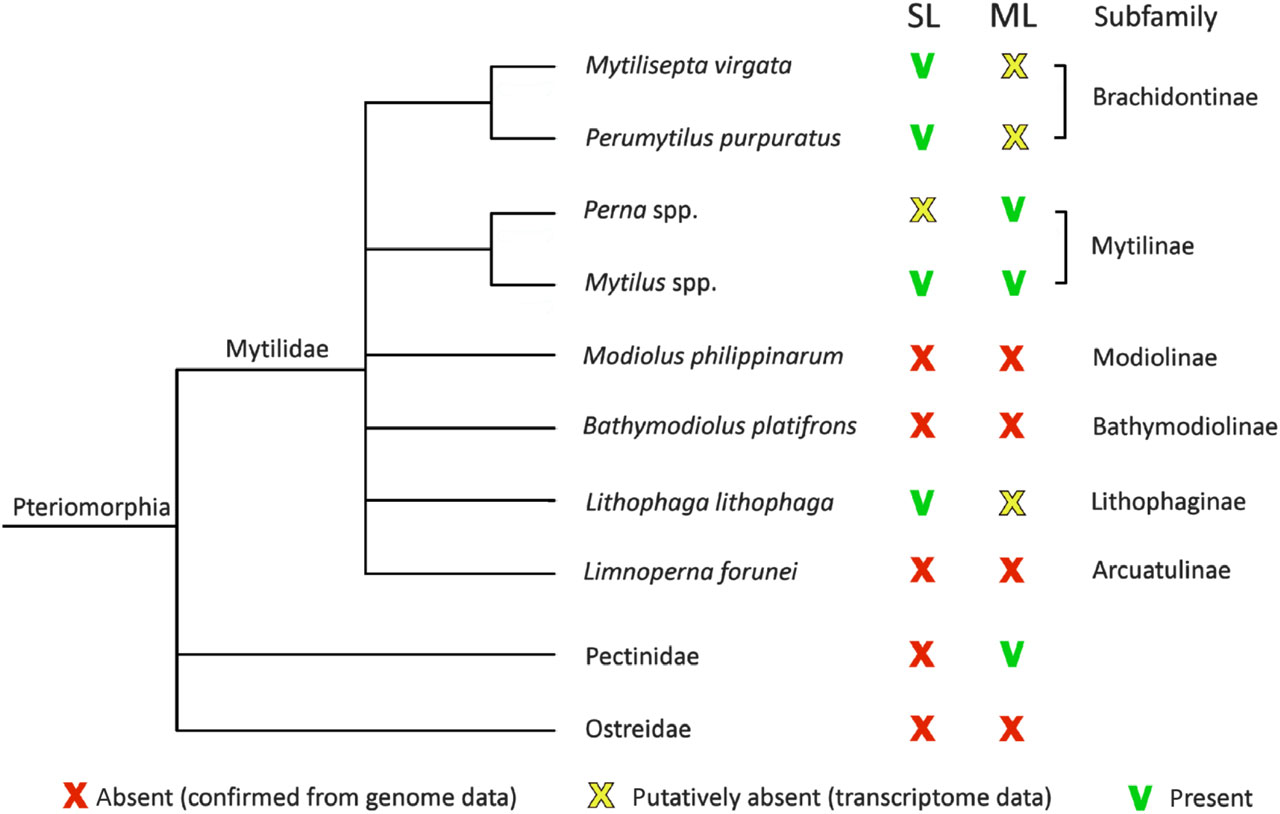
Comparative distribution of SeviL/R‐type (SL) lectin and mytilectin (ML) family members in bivalves. The presence of ML and SL was confirmed by genomic and/or transcriptomic data in the family Mytilidae, using Pectinidae, and Ostreidae as outgroups in the subclass Pteriomorphia. The cladogram was drawn based on the classification of bivalve species based on WoRMS data 33ription levels of SeviL in various *M. virgata* tissues, calculated *in silico* from RNA‐seq data in a pool of individuals and expressed as trascripts per milion (TPM) (panel A), or determined in single individuals with qRT/PCR (panel B) (see Materials and methods). Each bar represents the mean plus standard deviation of three technical replicates.

The distribution of SeviL‐like R‐type lectins and mytilectins in the different mytilid subfamilies is variable and partially overlapping (Fig. 4). Transcriptome analysis suggests that R‐type lectins (SeviL orthologues) are present in the subfamilies Brachidontinae (*Mytilisepta*, *Perumytilus*) and Lithophaginae (*Lithophaga*). On the other hand, both R‐type lectins and mytilectins were found in the transcriptomes of Mytilinae (*Mytilus*, *Perna*), and neither of the two lectin families were detected in the genomes of Modiolinae, *Bathymodiolinae* (deep‐sea mussels), or Arcuatulinae. While no SeviL‐like R‐type lectins could be found in the genomes of nonmytilid bivalves, mytilectin genes are present in Pectinidae (scallops). Overall, these observations reveal a markedly discontinuous distribution of SeviL‐like lectins and mytilectins in the bivalve tree of life, which suggests that their loss or retention may be dependent on unknown environmental or ecological factors.

### Expression of SeviL mRNA in *M. virgata* tissues

**Figure 5:**
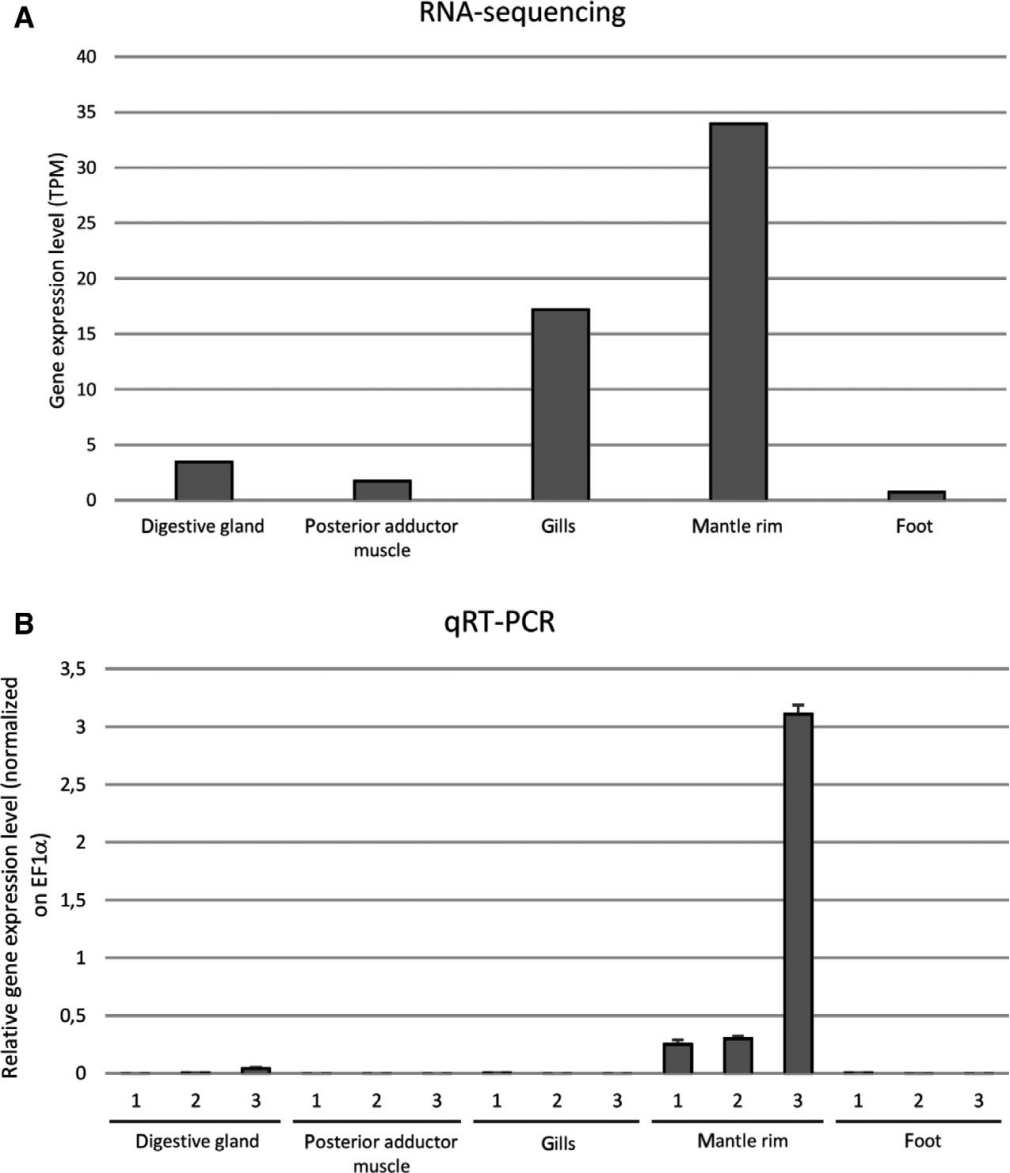
Transcription levels of SeviL in various M. virgata tissues, calculated in silico from RNA‐seq data in a pool of individuals and expressed as trascripts per milion (TPM) (panel A), or determined in single individuals with qRT/PCR (panel B) (see Materials and methods). Each bar represents the mean plus standard deviation of three technical replicates.

RNA‐seq mapping graphs based on various tissues collected from a pool of adult mussels (Fig. 5A) show high expression of SeviL (TPM > 10) in gills and mantle rim. SeviL expression levels were much lower in the digestive gland and posterior adductor muscle and barely detectable in foot (TPM < 1). The high expression of SeviL in mantle rim was confirmed by qRT/PCR on individual mussels (Fig. 5B). The specificity of expression of SeviL in tissues (gills and mantle) that are constantly exposed to the external environment suggests the possibility that this lectin is involved in recognition of glycans found on parasitic or symbiotic microorganisms.

### Glycan‐binding profile of SeviL

**Figure 6:**
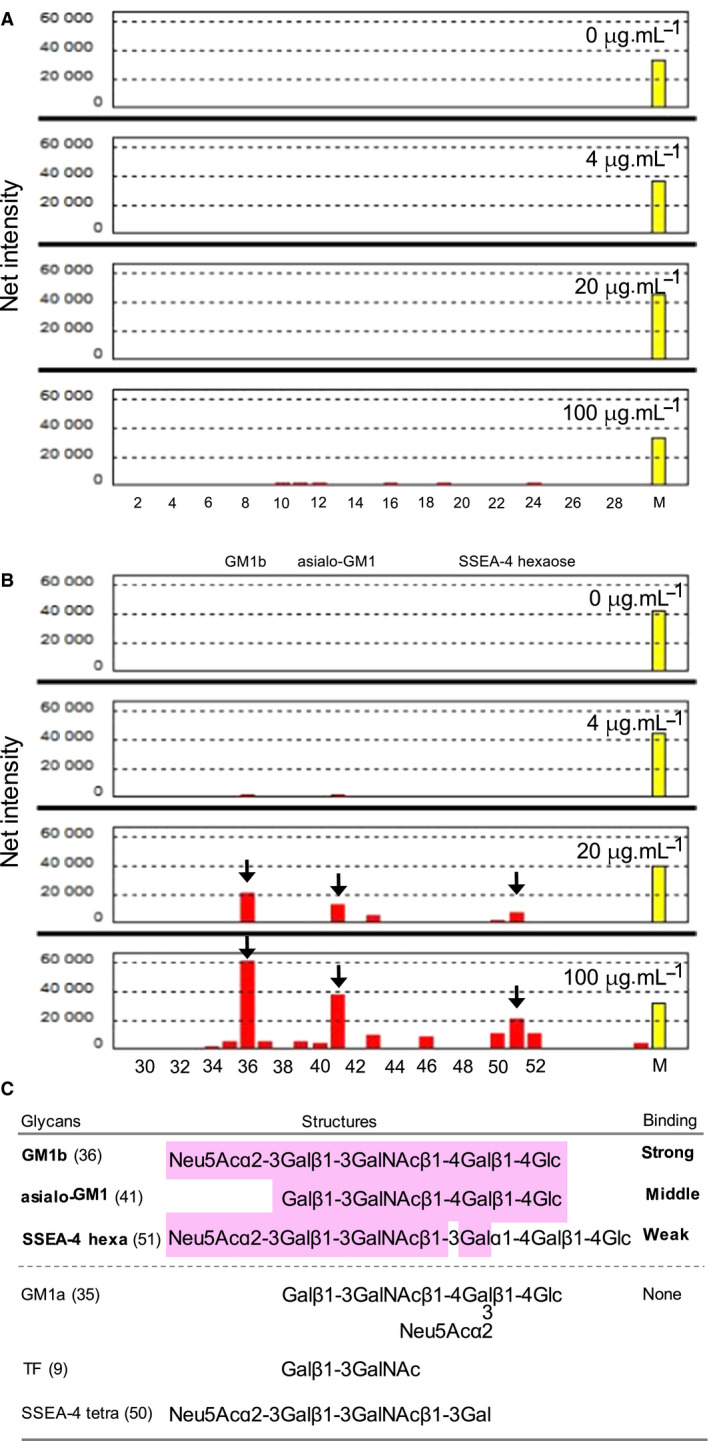
Glycan‐binding profile of SeviL. HyLite555‐labeled SeviL (0–100 μg·mL^−1^: right upper) was subjected to glycan array analysis combining glycan‐conjugated chips in which 52 glycan structures were immobilized and a surface plasmon resonance scanning detector (numbering as in Fig. S3A and Table S1). The evanescent‐field fluorescence occurring by the binding between HyLite555‐SeviL and the glycans (Fig. S3B) is represented as net intensities (*y*‐axis of these graphs). (A) The chip includes N‐glycans, O‐glycans, glycosaminoglycans, Lewis type oligosaccharides, derivatives of lactose and N‐acetyllactosamine and ABH‐type oligosaccharides (No. 1‐28 in Table S1). (B) The chip includes ganglio‐series oligosaccharides and globo‐series oligosaccharides (No. 29‐52 in Table S1). Arrows indicate positions (glycans number 36, 41 and 51) of glycans of GM1b, asialo‐GM1, and SSEA‐4 hexsaose, respectively. M. Internal standard marker for fluorescence. C. The structural motif of the glycans recognized by SeviL. The colored part highlights the structure shared by GM1b, asialo‐GM1 and SSEA‐4 hexa(ose), whose glycans are bound by SeviL (see panel B). GM1a, TF, and SSEA‐4 tetra(ose) are glycans that are not bound by the lectin.

The glycan‐binding profile of SeviL was determined by array analysis using 52 representative glycans, as illustrated and numbered in Fig. S3A and Table S1. SeviL bound significantly to the GM1b oligosaccharide (Neu5Acɑ2‐3Galβ1‐3GalNAcβ1‐4Galβ1‐4Glc; **36**) and its precursor asialo‐GM1 oligosaccharide (Galβ1‐3GalNAcβ1‐4Galβ1‐4Glc; **41**) (Fig. 6B and Fig. S3B). The lectin also interacted weakly with the glycan moiety of asialo‐GM2 (GalNAcβ1‐4Galβ1‐4Glc; **43**) oligosaccharide (which contains a GM1b or asialo‐GM1 oligosaccharide component) and the globo‐series SSEA‐4 hexaose (Neu5Acɑ2‐3Galβ1‐3GalNAcβ1‐3Galɑ1‐4Galβ1‐4Glc; **51**) (Fig. 6B). The total amount of SeviL bound with these three glycans was concentration‐dependent (Fig. 6B and Fig. S3B).

On the other hand, SeviL showed no notable binding to Thomsen–Friedenreich (TF‐) antigen (Galβ1‐3GalNAc; **9**) nor GM1a (Galβ1‐3GalNAcβ1‐4[Neu5Acα2‐3]Galβ1‐4Glc; **35**). SeviL displayed no significant interaction with N‐glycans (Fig. 6A: **1**–**7**), O‐glycans (Fig. 6A: **8** and **9**), and glycosaminoglycans (Fig. 6A: **10**–**14**), which are derived from glycoproteins.

### Asialo‐GM1 oligosaccharide‐dependent apoptosis

**Figure 7:**
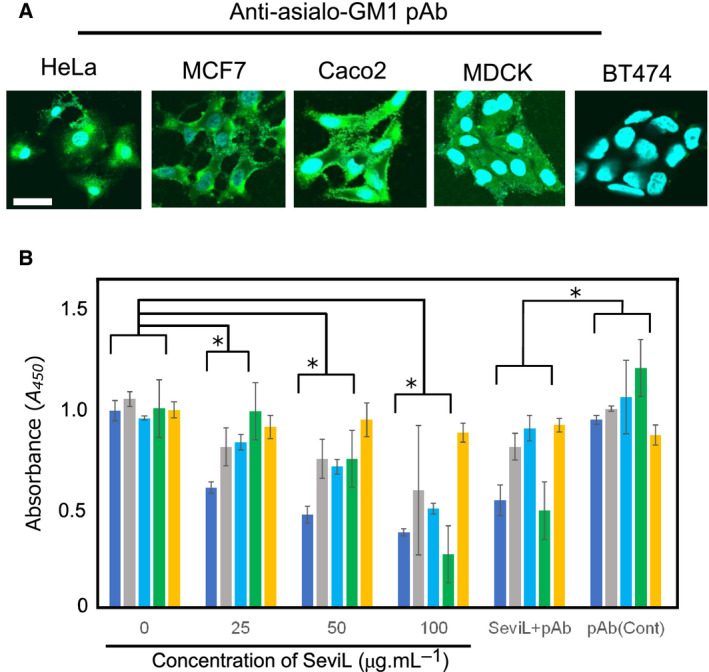
Detection of asialo‐GM1 expression on cell membranes by immunostaining. (A) Paraformaldehyde‐fixed cells were stained with anti‐asialo‐GM1 oligosaccharide pAb and AF488‐tagged goat anti‐rabbit Ab (green). Nuclei were counterstained with DAPI (blue). Magnification: ×40. Scale bar: 10 mm. (B) Cytotoxic effects of SeviL on human ovarian cancer (HeLa), breast cancer (MCF7, BT474), colonic cancer (Caco2), and dog kidney (MDCK) cells. Cells were treated with SeviL at various concentrations (0–100 μg·mL^−1^) for 24 h, and cell viability (expressed as A_450_; see M & M/ ‘Cell viability and cytotoxicity assays’) was determined by WST‐8 assay. SeviL + pAb: treatment with 50 μg·mL^−1^ SeviL plus anti‐asialo‐GM1 oligosaccharide polyclonal antibody (pAb). pAb (Cont): anti‐asialo‐GM1 oligosaccharide pAb without SeviL, as control. Data shown are mean ± SE (*n* = 3). *P* values (*P < 0.05) were obtained with Dunnett’s test.

Possible triggering of ganglioside‐dependent signals by SeviL was examined. Anti‐asialo‐GM1 pAb was applied to HeLa, MCF7, BT474, Caco2, and MDCK cells (see Materials and methods ‘Mussels, cell lines, and reagents’) and caused surface staining of each cell line except BT474 (Fig. 7A). Next, cells (10^5^ mL^−1^) were incubated with various concentrations of SeviL for 48 h, and cell viability and proportions of living cells were determined by WST‐8 assay. Increasing the SeviL concentration from 25 to 100 μg·mL^−1^ resulted in apoptosis (cell death) for HeLa, MCF7, Caco2, and MDCK (Fig. 7B), but not for BT474. Cotreatment with anti‐asialo‐GM1 pAb blocked the cytotoxic effect of SeviL (Fig. 7B, ‘SeviL + pAb’). These findings indicate that SeviL induced apoptosis mediated by ganglioside at the cell surface.

### Activation in HeLa cells of MAPK pathway and caspases

**Figure 8:**
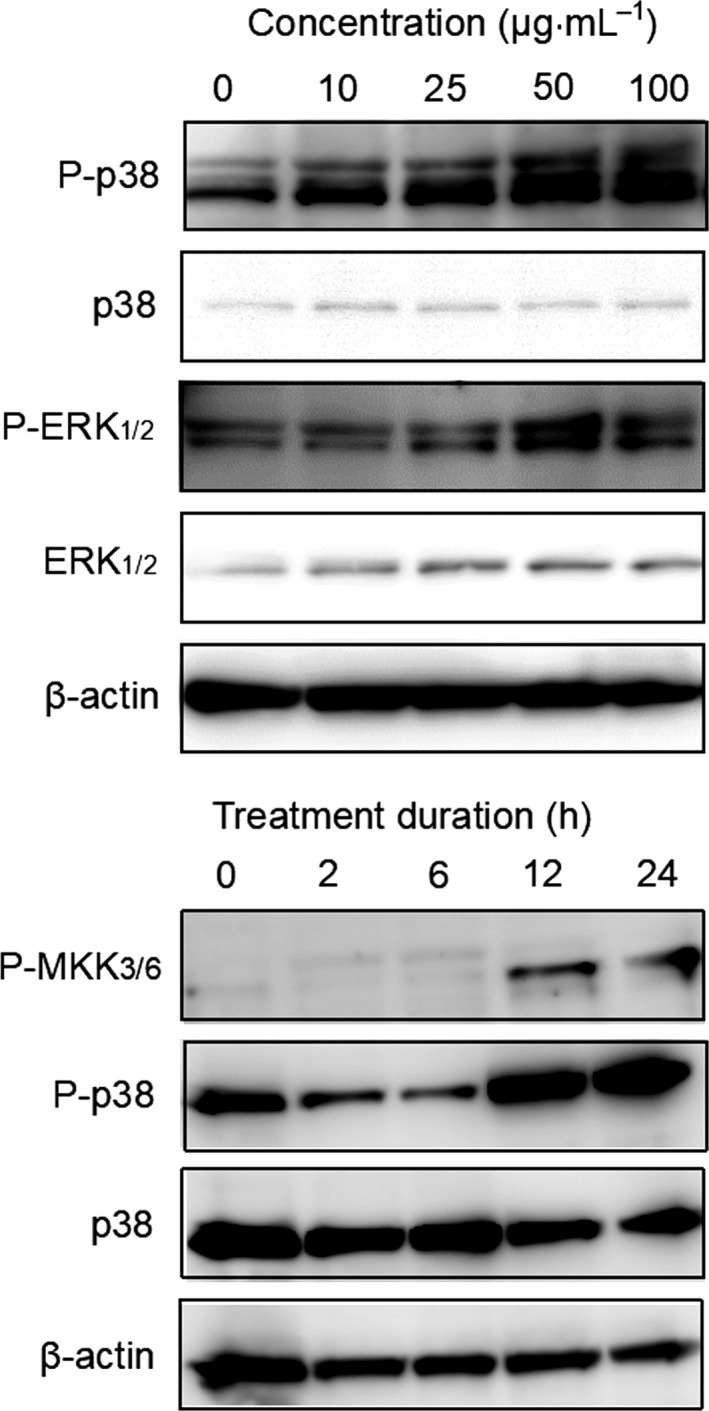
Phosphorylation of MAPKs by SeviL in HeLa cells. Cells (5 × 10^5^) were treated with SeviL at various concentration (0–100 μg·mL^−1^) or durations (0–24 h). Phosphorylation of kinases was evaluated by Western blotting of cell lysates. P‐p38, P‐ERK_1/2,_ and P‐MKK3/6: phosphorylated forms of p38, ERK_1/2_ and MKK3/6 kinase, respectively. MKK3/6 is the upstream kinase of ERK_1/2_.

**Figure 9:**
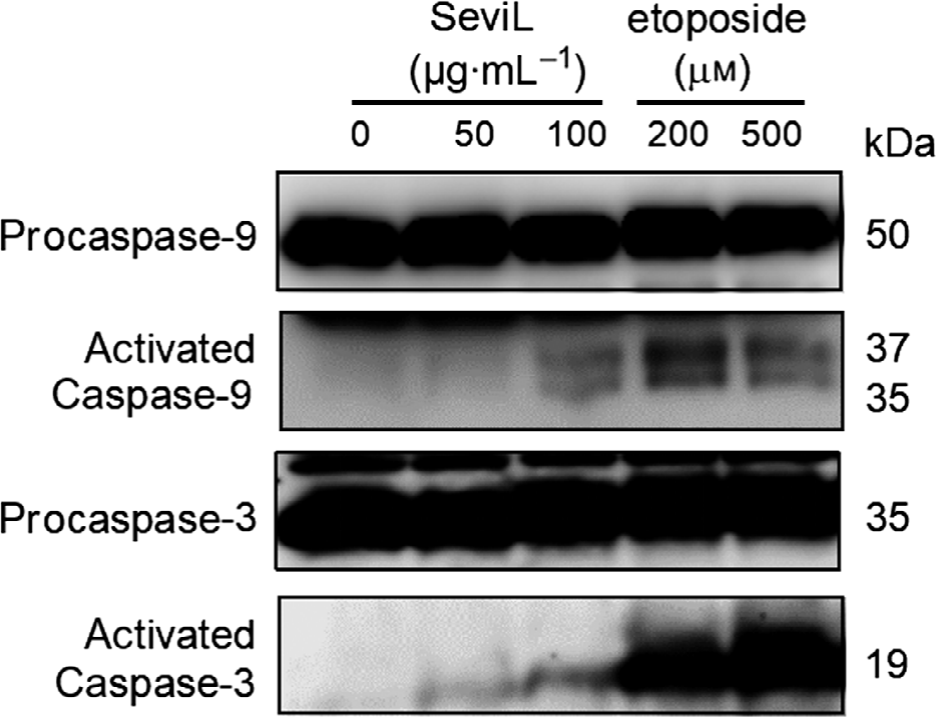
Induction of caspase‐3/9 cleavage by SeviL. HeLa cells were treated for 48 h with SeviL (50, 100 μg·mL^−1^), anticancer reagent etoposide (200, 500 μm) or lysates were prepared, and caspase‐3/9 activation was detected by Western blotting. Each experiment was performed in triplicate.

In HeLa cells, SeviL activated the MAPK pathway of extracellular signal‐regulated kinase (ERK)_1/2_ signaling cascade in dose‐dependent manner, as shown by Western blotting (Fig. 8, P‐ERK_1/2_ vs. ERK_1/2_). SeviL treatment also phosphorylated p38 mitogen‐activated protein kinase (Fig. 8, P‐p38 vs. p38) and activated caspase‐3/9 (Fig. 9, procaspase‐3 and 9 vs. activated caspase 3 and 9). These findings suggest that SeviL regulates cell physiological processes through similar MAPK pathways, including MEK/ERK and p38, and activate caspase‐3 via mitochondrial cycles.

### Localization of SeviL in *M. virgata* tissues

**Figure 10:**
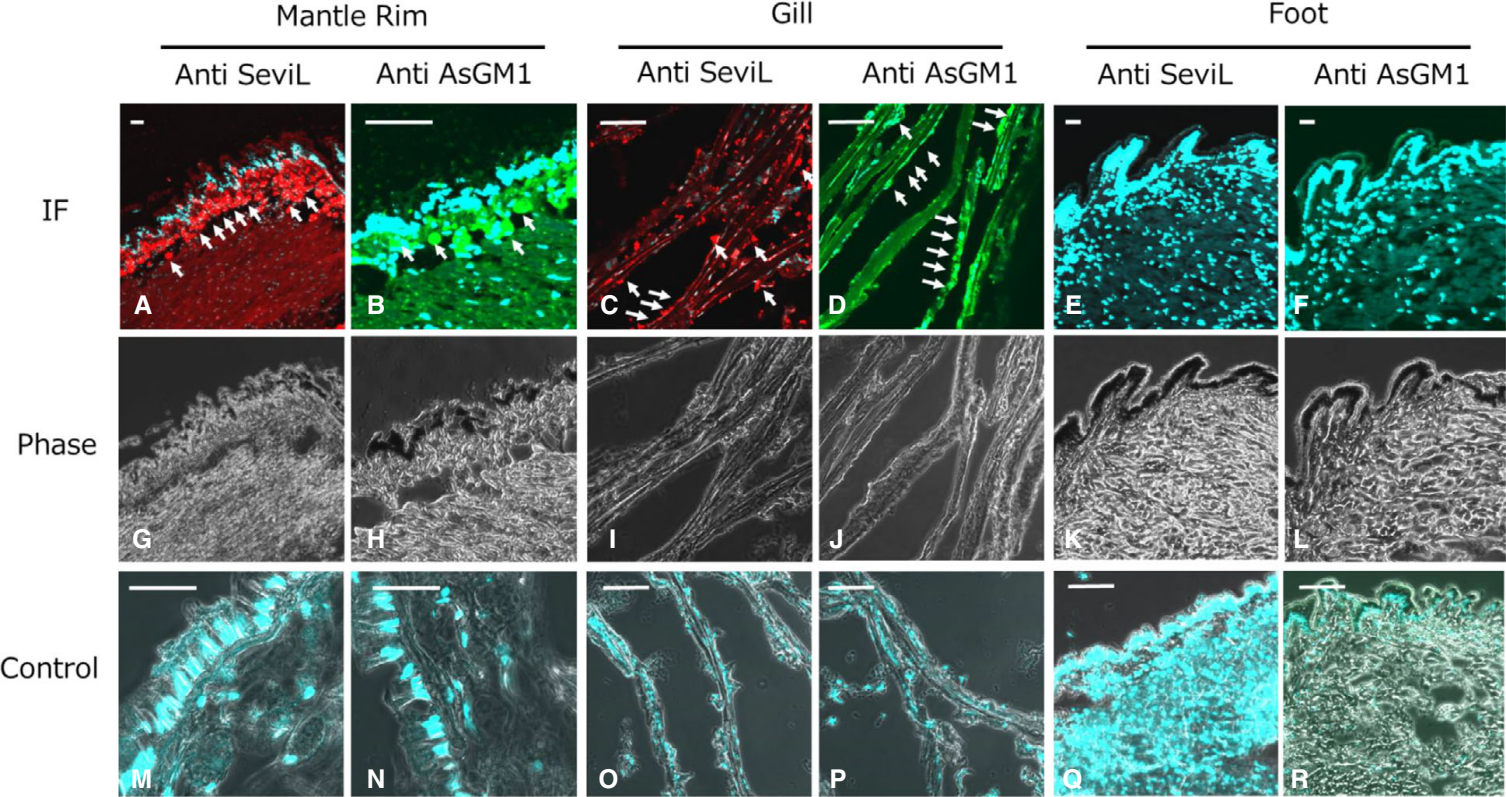
Localization of SeviL and substances which reacted with anti‐asialo‐GM1 polyclonal antibody in *M. virgata* tissues. Tissue sections derived from mantle rim, gill, and foot were applied by anti‐SeviL (column anti‐SeviL: A, C, E, G, I and K) and anti‐asialo‐GM1 (column Anti AsGM1: B, D, F, H, J, and I) polyclonal antibodies followed by Alexa 568 (red: for the detection Anti‐SeviL) or Alexa488 (green: for the detection of As asialo‐GM1) conjugated secondary anti‐rabbit IgG antibody. All sections were stained also with DAPI to detect nuclei (blule: A–F and M‐R). IF: immunofluoresence (A–F), phase: phase contrast (G–L) and control: applying nonimmune primary antibody (for SeviL: M, O, and Q) and without primary antibody (for asialo‐GM1 antibody reactant: N, P and R), respectively. Scale bars: 20 μm each.

SeviL signals detected by the antiserum indicated its specific presence in the outer part of the mantle rim and gills (Fig. 10A,C), but not in the foot (Fig. 10E). This localization pattern reflected the transcriptional levels of SeviL‐encoding mRNA coding in mussel tissues (Fig. 5A). The signals detected by the anti‐GM1 pAb showed the same pattern of distribution as the expression of SeviL (Fig. 10B,D). These findings suggest that the main sites of expression of SeviL in *M. virgata* match with the location of detection of the antigens detected by the asialo‐GM1 pAb. Since SeviL was obtained by the elution with the sugar‐containing buffer from the mantle and gills of the mussels during the purification, it seems reasonable for the lectin to be found in these tissues with binding its ligands (Table 1).

## Discussion

Over the past decade, glycobiological studies of nontraditional model organisms (such as bivalve mollusks) have revealed an unexpected diversity of lectins in various taxonomic groups. In this study, we have demonstrated the presence of an R‐type lectin (SeviL) in *Mytilisepta virgata*, a member of the family Mytilidae. This lectin family is characterized by a β‐trefoil fold structure and occurs across a wide range of animals, from microorganisms to humans. R‐type lectins have been reported previously from the invertebrate phyla Porifera 34, Annelida 35, and Echinodermata 36. SeviL was assigned to the R‐type lectin family on the basis of sequence similarities to the prototypical ricin B‐chain domain, but it displays features not found in other members of this family. First identified in a plant, R‐type lectins are found in a wide variety of organisms from bacteria to mammals, and numerous structures from this group have been analyzed in detail. Although a QxW motif is conserved in each of the three subdomains, no overall consensus sequence is found like that of C‐type lectins or galectins. The primary structure of SeviL shares less than 20% similarity with other invertebrate R‐type lectins (Fig. S2C), but there is much greater similarity (40–90%) among mytilid proteins (Fig. S2A). Both acidic and basic amino acids are found throughout the sequence of SeviL, in contrast to MytiLec‐1, which has acidic amino acids only on the C‐terminal side of each subdomain 12. Surprisingly, SeviL also possesses 6 hydrophobic amino acids at the C terminus, as found with MytiLec‐1 12. In the case of MytiLec‐1, these residues were essential for dimerization 13, but structural analysis will be needed to determine whether the same is true of SeviL.

Several similar sequences in other Mytilidae species besides *M. virgata* (Fig. 3) define a cluster of orthologues that we have named ‘SeviL‐like R‐type lectins’. Curiously, the taxonomic spread of these lectins only partially overlaps that of mytilectins 18. While no mytilectin was detected in the transcriptome of *M. virgata*, some mussel species (such as *Mytilus galloprovincialis*) possess both types of lectin, and others (such as *Modiolus philippinarum*) have neither (Fig. 4).

The transcription of *M. virgata* SeviL‐like lectin and *M. galloprovincialis* MytiLec‐1 was similarly confined to mantle and gills in both species (Fig. 5), suggesting that these lectins have similar roles in mussel physiology. However, while only the R‐type lectin family is expressed in *M. virgata*, both members of the R‐type family and the mytilectin family are encoded by the genome of *M. galloprovincialis*. It is presently unknown whether these two lectin families display an overlapping pattern of expression and are coregulated in this species. Determining how the expression of mussel lectins is modulated in response to external stimuli may bring new insights into the molecular ecology of these proteins, and helping to understand the role of lectin‐glycan interactions in the notable capacity of bivalve mollusks to adapt to new environments.

SeviL was purified by using a lactose‐conjugated affinity column, and its ability to hemagglutinate was inhibited in the presence of lactose and melibiose. However, such inhibition required a high concentration, 20 mm or more of the sugars, roughly 100 times the ligand concentration used in the glycan array analysis. This observation suggests that the binding by these disaccharides does not occur under physiological conditions.

SeviL, in same with an R‐type lectin from sea cucumber 36, requires divalent cations such as Ca^2+^ for its hemagglutination activity (Fig. S1A), whereas MytiLec‐1 shows no dependence on metal ions 12. In contrast to members of the mytilectin family, including MytiLec‐1 and CGL, which bind to α‐Gal in Gb3 13, 37, SeviL strongly binds to ganglio‐series GM1b oligosaccharide (Neu5Acα2‐3Galβ1‐3GalNAcβ1‐4Galβ1‐4Glc) and asialo‐GM1 (Galβ1‐3GalNAcβ1‐4Galβ1‐4Glc). SSEA‐4 hexaose (Neu5Acα2‐3Galβ1‐3GalNAcβ1‐3Galα1‐4Galβ1‐4Glc) and asialo‐GM2 (GalNAcβ1‐4Galβ1‐4Glc), which weakly interacted with SeviL, comprises a part of the structure of GM1b (Fig. 6B and Fig. S3B). On the other hand, SeviL did not bind to TF antigens (Galβ1‐3GalNAc), GM1a (Galβ1‐3GalNAcβ1‐4[Neu5Acα2‐3]Galβ1‐4Glc). These results suggest that Galβ1‐3GalNAcβ1‐4Galβ1‐4Glc is the core structure recognized by SeviL and that the presence of Neu5Ac at the nonreducing terminus is a desirable feature for sugar binding (Fig. 6C). Furthermore, the hydroxyl group at the C‐3 position of the 3rd Gal from the nonreducing terminus is required to be free (Fig. 6C). SeviL displayed a different carbohydrate‐binding specificity with GM1b and asialo‐GM1 compared with the cholera toxin, which bound to GM1a and fucosyl‐GM1 38. After all, this study found that this R‐type lectin of the mussels was glycosphingolipid glycan‐binding specific such as ganglioside and globoside, not but glycoprotein glycans (Fig. 6A).

The binding of SeviL to two ganglio‐series (GM1b and its precursor asialo‐GM1) and one globo‐series (SSEA‐4 hexaose) oligosaccharides is very interesting, since each glycan is expressed specifically by the target antigen for Guillain–Barré syndrome (GM1b) 39, natural killer cells and basophils (asialo‐GM1) 40, and glioblastoma multiforme (SSEA‐4) 41 in vertebrates, respectively. SeviL has potential clinical applications, similar to those of other invertebrate R‐type lectins that recognize specific glycans such as TF antigen (Galβ1‐3GalNAc) and LacdiNAc (GalNAcβ1‐4GlcNAc) 42, 43.

Exposure to SeviL led to increased metabolism and induction of apoptosis in mammalian cells bearing asialo‐GM1 oligosaccharide (Fig. 7), indicating the ability of the protein to regulate cell proliferation. SeviL is a dimer like MytiLec‐1 (Fig. 1B), whose self‐association is essential for cytotoxicity. The use of an anti‐asialo‐GM1 polyclonal antibody (pAb), which blocked the access to the target of SeviL, completely abrogated the effect of the lectin (Fig. 7B). The cell regulatory mechanisms triggered by the interaction between SeviL and GM1b oligosaccharides will also be clarified by using a specific anti‐GM1b antibody in future. SeviL activated various metabolic pathways (including MKK3/6, ERK_1/2_, p38, and caspase‐3/9). Both MytiLec‐1 and SeviL therefore can potentially regulate the growth of human cancer cells by binding their respective ligands and activating similar metabolic pathways (Figs 8 and 9). The mytilectin family is known to play roles not only in the regulation of cell death 44 and also cell proliferation 45, through the activation of kinases. SeviL and the other R‐type lectins isolated from Mytilidae may similarly have multiple activities, and it is possible that their expression may be regulated by signals external to the organism.

β‐Trefoil lectins of mussels have been proposed to be involved in defense against pathogenic microorganisms typically encountered by bivalves due to their filter‐feeding habits 16, 18, 46. By using immunohistochemistry techniques, the expression of SeviL was detected in tissues which are in direct contact with the internal and external environment (Fig. 10). The comparison between the transcriptional levels of SeviL‐like lectin and mytilectin in mussels grown in different environments may clarify the specific role of these lectins in immune defense against invading microorganisms. The spatial overlap between the signals detected with the anti‐asialo GM1 polyclonal antibody and the anti‐SeviL antibody (Fig. 10) suggests the presence of similar or identical antigens with asialo‐GM1 in the mussel tissues. The autoantibody which recognizes GM1b raised in patients affected by the Guillain–Barré syndrome arises from infection with Campylobacter, because specific lipopolysaccharides of the bacteria possess structures similar to gangliosides 39. This evidence may support the hypothesis that the glycans recognized by SeviL may be present both in invading microorganisms and in the tissues of species pertaining to the family Mytilidae. It has been recently reported that the lectin subunit of the cholera toxin, which is known to bind GM1a, also binds to lipooligosaccharides and is capable of inhibiting the growth of genus *Campylobacter* 47. The glycan structures of most invertebrates and microorganisms remain to be thoroughly investigated 48, 49, and the application of structural glycobiology approaches to these phyla will provide a significant improvement in the knowledge on this subject.

Besides the immune response of bivalves in response to infection by pathogenic microorganisms, the study of the infection and defense mechanisms enacted by these marine bivalves against neoplasia has also met a considerable interest in recent years. The group of Goff showed that horizontal transmission of cancer cells in bivalves resulted from activation of the retro‐transposon gene ‘Steamer’ 50. Such neoplastic cells may be propagated from one individual and transmitted to others through sea water 51. In recent, the group of Metzger elucidated how the cancer cells of mussels were transferred across the Atlantic and Pacific Oceans and between the Northern and Southern hemispheres 52. By knowing this situation, we will have more interest in how the cancer cells transfer into the mussels in the Asian area. In order to better elucidate the physiological role of SeviL‐like lectins, one of our next goals will be to investigate whether their administration to tumor cells derived from bivalves may have a significant effect on cell growth regulation.

SeviL and MytiLec‐1 bind to β‐ and α‐galactosides, respectively, but it is not yet certain whether the natural ligand of these proteins is found within the organism itself or in the surrounding environment. The physiological roles of these proteins will remain unclear, however, until their target glycans are identified. Certain mollusks have characteristic glycosphingolipids with Gal or GalNAc at their glycan termini 53, 54. Such glycans are potential ligands for mytilectins, and similar ligands may exist for SeviL as well. Although the protein appears from its sequence to be a β‐trefoil, it shows limited conservation to other such proteins at the sugar‐binding sites (Fig. S4). Characteristics of the molecular structure of the protein may help understand the function of lectins by revealing the modes of endogenous and exogenous ligand binding. Although the history of lectins in the mollusks is so long, the studies on the functional characterization of the β‐trefoil fold lectin in the bivalves have just begun.

## Materials and methods

### Mussels, cell lines, and reagents

Mussels (*M. virgata*) were collected from the seashore at Saikai City, Nagasaki prefecture, Japan, under the permission of the Saikai‐Oosaki fisheries union. The shells were removed, and bodies were stored whole at −80 °C. Human cell lines HeLa (cervical cancer), MCF7 and BT474 (breast cancer), and Caco2 (colonic cancer) were from American Type Culture Collection (ATCC). Dog kidney cell line MDCK was kindly provided by T. Fujiwara. Lactose, melibiose, sucrose, D‐Gal, D‐Glc, D‐Man, D‐Fuc, D‐GalNAc, D‐GlcNAc, standard protein markers for SDS/PAGE, porcine stomach mucin, fetuin, and penicillin–streptomycin for cell culture were from FUJIFILM Wako Pure Chemical Corp. (Osaka, Japan). Bovine submaxillary mucin was from MP Biomedical (Illkirch‐Graffenstaden, France). Lactosyl‐agarose gel was from EY Laboratories (San Mateo, CA, USA). RPMI 1640 medium and fetal bovine serum (FBS) were from Thermo Fisher Scientific (Waltham, MA, USA). Cell Counting Kit‐8 including 2‐(2‐methoxy‐4‐nitrophenyl)‐3‐(4‐nitrophenyl)‐5‐(2,4‐disulfophenyl)‐2H‐tetrazolium) monosodium salt (WST‐8), HiLyte Fluor 555 labeling kit‐NH_2,_ and Biotin Labeling kit‐NH_2_ were from Dojindo Laboratories (Kumamoto, Japan). HRP‐conjugated β‐actin monoclonal antibody (mAb) was from FUJIFILM. Anti‐asialo‐GM1 polyclonal antibody (pAb) (rabbit immunoglobulin) was from Cedarlane (Ontario, Canada). Anti‐p38, antiphosphorylated p38 (pT180/pY182), anti‐Erk1, and antiphosphorylated ERK_1/2_ (pT202/pY204) monoclonal antibodies (mAbs) were from Becton Dickinson (Franklin Lakes, NJ, USA). Antiphosphorylated MKK3/6 (MKK3(S189)6(S207) 22A8) and anticaspase‐9 mAbs were from Medical and Biological Laboratories (Nagoya, Japan). Anticaspase‐3 mAb was from Cell Signaling Technology (Danvers, MA, USA). HRP‐conjugated anti‐mouse or anti‐rabbit IgG as secondary antibody was from Chemicon International (Temecula, CA, USA). Alexa Fluor**^®^** 568‐labeled goat anti‐rabbit IgG and Alexa Fluor**^®^** 488‐labeled goat anti‐rabbit IgG were from Abcam (Cambridge, UK). Can Get Signal^®^ Immunoreaction Enhancer Solution 1 and 2 were from Toyobo Co. (Osaka, Japan). Tissue‐tek compound was from Sakura Finetek Co (Tokyo, Japan). Low fluorescence and silicon‐coated glass slides were from Matsunami Glass Industry (Kishiwada, Osaka, Japan).

### Lectin purification

Mussel mantles and gills were homogenized with 10 volumes (w/v) 150 mm NaCl containing 10 mm Tris/HCl, pH 7.5 (TBS) with 10 mm CaCl_2_. Supernatant (Sup 1) was collected by centrifugation at 27 500 ***g*** for 1 h at 4 °C as described in our previous study 12, with some modification. Precipitate was homogenized with 10 volumes (w/v) TBS containing 50 mm lactose, and supernatant (Sup 2) was collected as above. Sup 2 was dialyzed extensively against TBS. Both Sup 1 and Sup 2 were applied to lactosyl‐agarose column (5.0 mL), and the column was washed with TBS until absorbance at 280 nm (A_280_) of effluent reached baseline level. Lectin was eluted with TBS containing 50 mm lactose.

### Hemagglutination assay and sugar‐binding specificity assay

Hemagglutination assay was performed in 96‐well V‐shaped plates as described previously 55. Twenty microliters of twofold dilution of purified lectin in TBS was mixed with 20 μL of a 1% suspension (with TBS; v/v) of trypsinized, glutaraldehyde‐fixed rabbit erythrocytes, TBS, or TBS with 0.2% Triton X‐100. Plates were incubated for 1 h at room temp, and formation of a sheet (agglutination‐positive) or dot (agglutination‐negative) was observed and scored as lectin titer. For sugar‐binding specificity assay, 20 μL of sugar solution (200 mm) was serially diluted with TBS and mixed with 20 μL of lectin solution (adjusted to titer 16), trypsinized/glutaraldehyde‐fixed rabbit erythrocytes, or TBS containing 1% Triton X‐100. Plates were incubated for 1 h at room temp, and minimal inhibitory sugar concentration was determined.

### Protein quantification and molecular mass determination

Protein was quantified using a protein assay kit (Thermo Fisher/ Pierce) based on the principle of bicinchoninic acid for colorimetric detection 56, 57, using ovalbumin as standard. SDS/PAGE 58 was performed in 15% (w/v) acrylamide gel under reducing or nonreducing conditions, and gels were stained by Coomassie Brilliant Blue R‐250.

### Analytical ultracentrifugation

Sample concentration was estimated as 1.0 mg·mL^−1^ from A280 measurement. Sedimentation velocity experiments were performed using an Optima XL‐I analytical ultracentrifuge (Beckman Coulter, Brea, CA, USA) with An‐50 Ti rotor. Analytical cells (with standard Epon two‐channel centerpiece and sapphire windows) were loaded with 400 μL sample and 420 μL reference solution (50 mm potassium phosphate, pH 7.4, 0.1 m NaCl). Prior to each run, the rotor was kept stationary at 293 K in vacuum chamber for 1 h for temperature equilibration. A280 scans were performed at 10‐min intervals during sedimentation at 201 600 ***g*** and analyzed using the continuous distribution (c(s)) analysis module in SEDFIT 59. Frictional ratio (*f*/*f*_0_) was allowed to float during fitting. c(s) distribution was converted to molar mass distribution c(M). Partial specific volume of protein, solvent density, and solvent viscosity were calculated from standard tables using the program SEDNTERP 60.

### Determination of primary structure of SeviL by mass spectrometry

The partial peptide sequence of SeviL was derived by Proteomics International (Nedlands, WA, Australia). 200 μg lectin was dialyzed extensively against distilled water to remove salt, lyophilized, and digested by trypsin, and peptides were extracted by standard techniques 61. Peptides were analyzed by electrospray ionization mass spectrometry using Prominence nano HPLC system (Shimadzu, Kyoto, Japan) coupled to 5600 triple time‐of‐flight (TOF) mass spectrometer (AB Sciex, Framingham, MA, USA). Tryptic peptides were loaded onto Zorbax 300SB‐C18 column, 3.5 mm (Agilent, Santa Clara, CA, USA) and separated on a linear gradient of water/ acetonitrile/ 0.1% formic acid (v/v). MS/MS spectra were analyzed using PEAKS Studio software platform v. 4.5 SP2 (Bioinformatics Solutions, Waterloo, ON, Canada) with manual interpretation.

### Transcriptomic analysis of full‐length cDNA

cDNA sequence of SeviL obtained as above was used to screen *de novo‐*assembled transcriptome data of *M. virgata* obtained from four tissues (i.e., gills, mantle rim, posterior adductor muscle, and digestive gland) from a pool of mussels collected at the seashore of Saikai city 29.

The assembled contig corresponding to the putative mRNA lectin sequence was identified by tBLASTn (e‐value threshold was set at 0.05). The partial peptides sequences obtained as described in the previous section were used as queries for BLAST searches against the transcriptome. Correct assembly of the consensus transcript was confirmed by back‐mapping RNA‐seq reads to the sequence and by assessment of uniform and homogenous mapping along the entire coding sequence. The expression level of SeviL in various tissues was calculated *in silico* as TPM (transcripts per million) using CLC Genomics Workbench v.10 RNA‐seq mapping tool (Qiagen, Hilden, Germany), setting length fraction parameter to 0.75, similarity fraction parameter to 0.98, and match/mismatch/deletion penalties to 3/3/3. RNA‐seq datasets from the tissues mentioned above were used for this analysis 29.

To further confirm the tissue specificity of SeviL, qRT/PCR analyses were carried out on three individual mussels, as described in 29. In this case, the sequence‐specific primers designed for SeviL are (5′ ‐> 3′): AATTTGGGGCGTAAAGACCT (forward primer) and GGACTCTCTTCCGAGGGTG (reverse), aiming at the amplification of a 111‐bp target sequence.

### Sequence data availability

The cDNA sequence of SeviL, SeviL‐2, and the orthologue sequences identified in publicly available transcriptomes of other mytilid species have been deposited in the GenBank repository, under the accession numbers MK434191–MK434201. The sequence alignments among each orthologue in the different species or the subdomains in the polypeptide are analyzed by using the MUSCLE program (http://www.drive5.com/muscle) 31.

### Glycan array analysis

Glycan array analysis was performed by Sumitomo Bakelite Co. (Tokyo, Japan). SeviL was fluorescence‐labeled (λ_ex/em_ 555/570 nm) using HiLyte Fluor 555 labeling kit‐NH_2_ as per the manufacturer’s instructions. A wide range of 52 glycans including N‐glycans, O‐glycans, Lewis glycans, lactosamine, blood‐type glycans, gangliosides, and globosides were immobilized on wells. Fluorescence‐labeled SeviL at concentrations ranging from 0 to 100 μg·mL^−1^ were incubated overnight at 4 °C with shielding from light. SeviL‐binding glycans were detected by using Bio‐REX Scan 300, an evanescent fluorescence scanner (Rexxam Co. Ltd., Osaka, Japan). Wavelength of laser light was used for Cy3, and the exposure time was 300 ms 62.

### Cell viability and cytotoxicity assays

Cells were maintained in RPMI 1640 supplemented with heat‐inactivated FBS 10% (v/v), penicillin (100 IU·mL^−1^), and streptomycin (100 μg·mL^−1^) at 37 °C. Cytotoxic effects and cell growth following treatment with SeviL at concentrations ranging from 0 to 100 μg·mL^−1^ were determined using Cell Counting Kit‐8 containing WST‐8 12. Cells (2 × 10^4^, in 90 μL solution) were seeded into 96‐well flat‐bottom plates and treated with 10 μL lectin for 24 h at 37 °C. To evaluate glycan‐inhibitory effects, anti‐asialo‐GM1 oligosaccharide pAb (50 μg·mL^−1^) was co‐incubated with cells in addition to lectin for 24 h and then applied to the assay system. For assay of effect on cell growth, each well was added with 10 μL WST‐8 solution and incubated 4 h at 37 °C. Cell survival rate was determined by measuring A_450_ (reference: A_600_) with a microplate reader (model iMark; Bio‐Rad, Tokyo, Japan).

### Detection of activated signal transduction molecules and their phosphorylated forms

HeLa cells (5 × 10^5^) were cultured with SeviL (0–100 μg·mL^−1^) for 24 h and then lysed in 200 μL RIPA buffer. Lysate was separated by SDS/PAGE and electrotransferred onto PVDF membrane as described previously 63. Primary mAbs used were directed to p38 (1:3000; mouse), phospho‐p38 (1:3000; mouse), ERK_1_ (1:3000; mouse), phospho‐ERK_1/2_ (1:3000; mouse), phospho‐MKK3/6 (1:3000; mouse), caspase‐3 (1:5000; rabbit), and caspase‐9 (1:5000; mouse). These antibodies were applied in Can Get Signal solution 1. Membrane was incubated for 24 h at 4 °C. HRP‐conjugated secondary antibody was diluted 1:5000 in Can Get Signal solution 2 64.

### Immunocytochemical analysis of asialo‐GM1 oligosaccharide expression

Cells (1 × 10^6^) were fixed with 4% paraformaldehyde in PBS for 15 min at room temperature, washed 3× with PBS, blocked with 1% BSA in PBS for 30 min at room temperature. They were washed 3× with PBS, incubated with or without 100 μL anti‐asialo‐GM1 oligosaccharide pAb (dilution 1:200 with PBS) at 4 °C for 30 min, washed 3× with PBS, treated with 100 μL Alexa Fluor^®^ 488‐tagged goat anti‐rabbit IgG (dilution 1:200 in PBS) at 4 °C for 30 min. Cells were placed onto low fluorescence glass slides, mounted with 50% glycerol solution, and examined by confocal microscopy. Confocal images were obtained using FV10i FLUOVIEW (Olympus, Tokyo, Japan).

### Immunohistochemistry of SeviL expressions on the mussel tissues

Mussel organs (gill, mantle rim, and foot) were cut into around 1‐cm‐square pieces, embedded in the Tissue‐tek compound and frozen in isopentane, cooled in by liquid nitrogen. The frozen tissue block was sliced on 6‐μm‐thick with Leitz cryostat (Leica Instruments, Nussloch, Germany), placed on silicon‐coated glass slides. Sections were sequentially fixed in PBS containing 4% paraformaldehyde for 15 min at room temperature, incubated in blocking solution containing 0.05% saponin and 1% BSA in PBS for 30 min, incubated with or without anti‐SeviL or anti‐asialo‐GM1 oligosaccharide pAb (dilution 1:100 with blocking solution) at room temperature for 1 h. After washing the tissues with PBS, they were treated with Alexa Fluor^®^ 568‐labeled goat anti‐rabbit IgG or Alexa Fluor^®^ 488‐labeled goat anti‐rabbit IgG (dilution 1:100 in blocking solution) at room temperature for 1 h, washed with PBS, mounted with 50% glycerol solution, and observed by confocal microscopy FV10i FLUOVIEW. Nuclei were stained by DAPI (364/454 nm) 64.

### Statistical analysis

Experiments were performed in triplicate, and results presented as mean ± standard error (SE). Data were subjected to one‐way analysis of variance (ANOVA) followed by Dunnett's test, using spss statistics software package, v. 10 (www.ibm.com/products/spss-statistics). Differences with *P* < 0.05 were considered significant.

## Conflict of interest

The authors declare no conflict of interest.

## Author contributions

YF, MG, JRHT, and YO designed the study; YF, MG, FS, KK, IH, SR, SMAK HF, and YO performed the experiments; JRHT, YOg, MH, and AP contributed new reagents/analytic tools; YF, MG, KK, SS, YK, TY, YOg, and YO analyzed data; YF, YO, MG, and JRHT wrote the paper.

## Supporting information


**Fig. S1.** Calcium‐dependent hemagglutination and *de novo* sequence of SeviL.
**Fig. S2.** Pairwise sequence comparison of SeviL, and schematic organization of *M. galloprovincialis* locus encoding of SeviL‐like lectin.
**Fig. S3.** Glycan‐array analysis.
**Fig. S4.** A homology model of SeviL.
**Table S1.** List of 52 oligosaccharides used for the glycan‐array analysis.
