# Supplementary material for: A GM1b/asialo‐GM1 oligosaccharide‐binding R‐type lectin from purplish bifurcate mussels Mytilisepta virgata and its effect on MAP kinases
Source: FEBS J. 2019 Dec 24;287(12):2612–30. doi: 10.1111/febs.15154 (PMC7317968; doi:10.1111/febs.15154)
Supplement: Supplementary file 1 — Fig. S1. Calcium‐dependent hemagglutination and de novo sequence of SeviL. Fig. S2. Pairwise sequence comparison of SeviL, and schematic organization of M. galloprovincialis locus encoding of SeviL‐like lectin. Fig. S3. Glycan‐array analysis. Fig. S4. A homology model of SeviL. Table S1. List of 52 oligosaccharides used for the glycan‐array analysis. [file FEBS-287-2612-s001.zip › febs15154-sup-0001-SupInfo.pdf]

The FEBS Journal

www.febsjournal.org

A GM1b/asialo-GM1 oligosaccharide-binding R-type lectin from purplish bifurcate mussels *Mytilisepta virgata* and its effect on MAP kinases

Yuki Fujii, Marco Gerdol, Sarkar M. A. Kawsar, Imtiaj Hasan, Francesca Spazzali, Tatsusada Yoshida, Yukiko Ogawa, Sultana Rajia, Kenichi Kamata, Yasuhiro Koide, Shigeki Sugawara, Masahiro Hosono, Jeremy R. H. Tame, Hideaki Fujita, Alberto Pallavicini and Yasuhiro Ozeki

DOI: 10.1111/febs.15154

A GM1b/asialo-GM1 oligosaccharide-binding R-type lectin from purplish bifurcate mussels *Mytilisepta virgata* and its effect on MAP kinases

Fujii Y, Gerdol M, Kawsar SMA, Hasan I, Spazzali F, Yoshida T, Ogawa Y, Rajia S, Kamata K, Koide Y, Sugawara S, Hosono M, Tame JRH, Fujita H, Pallavicini A, Ozeki Y.

Suppl Fig S1

A.

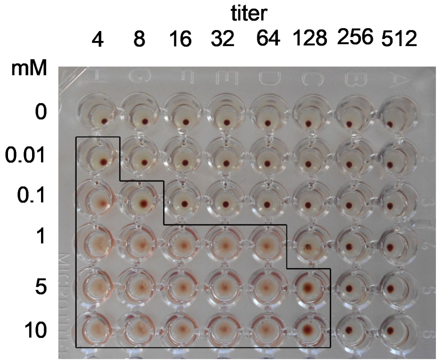

B.

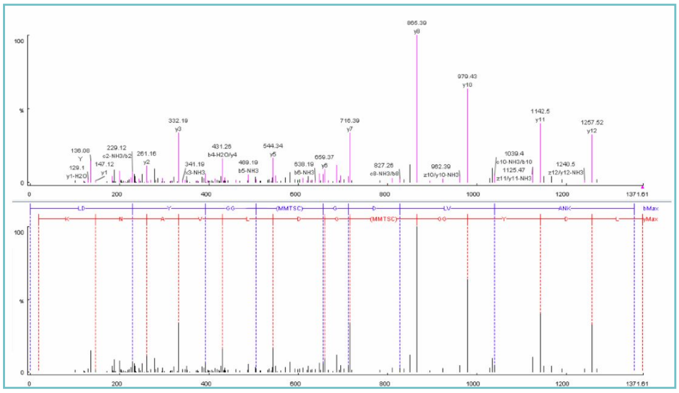

C.

```
                                           20                                   40
                                            |                                    |
SeviL    MSSVTIGKCY  IQNRENGGRA  FYNLGRKDLG  IFTGKMYDDQ
SeviL-2  MSSVTIGKCY  IQNRENGGRA  FYNLGRKDLG  IFAGKMYDDQ

                                           60                                   80
                                            |                                    |
SeviL    IWSFQKSDTP  GYYTIGRESK  FLQYNGEQVI  MSDIEQDTTL
SeviL-2  IWTFQKSDTP  GYYTTGRESK  FLQYDGKQVR  MSDIQQDNTL

                                          100                                  120
                                            |                                    |
SeviL    WSLEEVPEDK  GFYRLLNKVH  KAYLDYNGGD  LVANKHQTES
SeviL-2  WSLEEVPEDK  GFSRLLNKVH  KAYLDYNGGD  LVANKHQTES

SeviL    EKWILFKAY
SeviL-2  EKWIWFKAY
```

Suppl Fig S1. **Calcium-dependent hemagglutination and *de novo* sequence of SeviL.**
A. Calcium dependent hemagglutination of SeviL. Left: concentration of calcium chloride (mM). Top: titer of hemagglutinin of SeviL. After serial dilution of SeviL (10 μg/mL at the beginning) from left to right, each concentration calcium chloride was added in the wells. Box: positive of hemagglutination by SeviL.

B. *de novo* sequence of trypsinized peptide m/z 685.81 $(MH_2)^{2+}$ was identified by quadropore mass spectrometry of SeviL (^{104}Leu to ^{115} Lys in Fig 2A).

C. Protein sequence alignment between *M. virgata* SeviL and Sevil-2. The differences between the two proteins are at (respectively) locations 33 (Thr vs. Ala), 43 (Ser vs. Thr), 55 (Ile vs. Thr), 65 (Asn vs. Asp), 67 (Glu vs. Lys), 70 (Ile vs. Arg), 75 (Glu vs. Gln), 78 (Thr vs. Asn), 93 (Tyr vs. Ser), and 125 (Leu vs. Trp) (Fig. 3: mytvir1 vs. mytvir2).

Suppl Fig S2

A.

		1	2	3	4	5	6	7	8	9	10	11
mytgal1	1		99.24	98.47	93.13	95.42	38.81	38.81	33.08	30.30	34.53	39.71
mytgal2	2	99.24		99.24	92.37	94.66	38.81	38.81	33.83	30.30	34.53	40.44
mytedu	3	98.47	99.24		92.37	94.66	38.81	38.81	33.83	30.30	34.53	40.44
mytcal	4	93.13	92.37	92.37		94.66	39.55	39.55	33.83	31.06	35.25	41.18
myttro	5	95.42	94.66	94.66	94.66		37.31	37.31	30.83	28.03	34.53	40.44
mytvir1	6	38.81	38.81	38.81	39.55	37.31		92.25	51.13	51.15	32.14	33.33
mytvir2	7	38.81	38.81	38.81	39.55	37.31	92.25		48.87	48.85	32.86	31.85
perpur1	8	33.08	33.83	33.83	33.83	30.83	51.13	48.87		36.09	29.20	25.93
perpur2	9	30.30	30.30	30.30	31.06	28.03	51.15	48.85	36.09		28.57	25.93
litlit2	10	34.53	34.53	34.53	35.25	34.53	32.14	32.86	29.20	28.57		33.57
litlit1	11	39.71	40.44	40.44	41.18	40.44	33.33	31.85	25.93	25.93	33.57	

B.

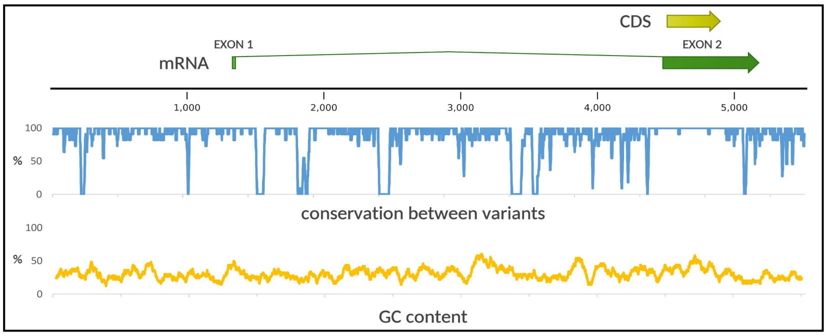

C.

R-type lectin family	Organisms		1	2	3	4	5	6	7	8	9	10
SeviL [1-129]	invertebrate	1		19.71	19.46	18.57	17.12	16.78	16.15	16.03	15.67	13.24
cytolysin [P19247_338-465]	bacteria	2	19.71		19.21	19.57	18.62	12.67	22.31	19.55	21.74	18.94
pierisin [Q9U8Q4_413-560]	invertebrate	3	19.46	19.21		17.45	13.82	14.47	13.25	17.57	18.79	14.19
CEL-III [Q868M7_115-245]	invertebrate	4	18.57	19.57	17.45		17.93	15.54	27.48	16.67	19.12	20.00
Sponge [P28586_1-144]	invertebrate	5	17.12	18.62	13.82	17.93		15.89	16.67	14.58	16.89	13.33
BEL [R4GRU6_1-146]	fungus	6	16.78	12.67	14.47	15.54	15.89		19.86	15.54	21.48	15.65
Achn [Q9KWN0_45-160]	bacteria	7	16.15	22.31	13.25	27.48	16.67	19.86		24.19	27.69	18.40
Mrc1 [Q61830_22-142]	manmmal	8	16.03	19.55	17.57	16.67	14.58	15.54	24.19		12.40	16.92
Ricin B [P02879_321-448]	plant	9	15.67	21.74	18.79	19.12	16.89	21.48	27.69	12.40		17.56
EW29 [O96048_3-127]	invertebrate	10	13.24	18.94	14.19	20.00	13.33	15.65	18.40	16.92	17.56	

Suppl Fig S2. Pairwise sequence comparison of SeviL, and schematic organization of *M. galloprovincialis* locus encoding of SeviL-like lectin.

A. Identity matrix in pairwise sequence comparison between SeviL-like R-type lectin protein sequences from Mytilidae.

B. Schematic organization of the *M. galloprovincialis* locus encoding the SeviL-like lectin mytgal1 (see Fig. 3). The sequence conservation, at the nucleotide level, with the paralogous gene encoding mytgal2 is reported, together with genomic GC content. mRNA and coding sequence (CDS) annotations are displayed.

C. Identity matrix in pairwise sequence comparison between SeviL and R-type lectin protein sequences from various organisms.

Suppl Fig S3

A.

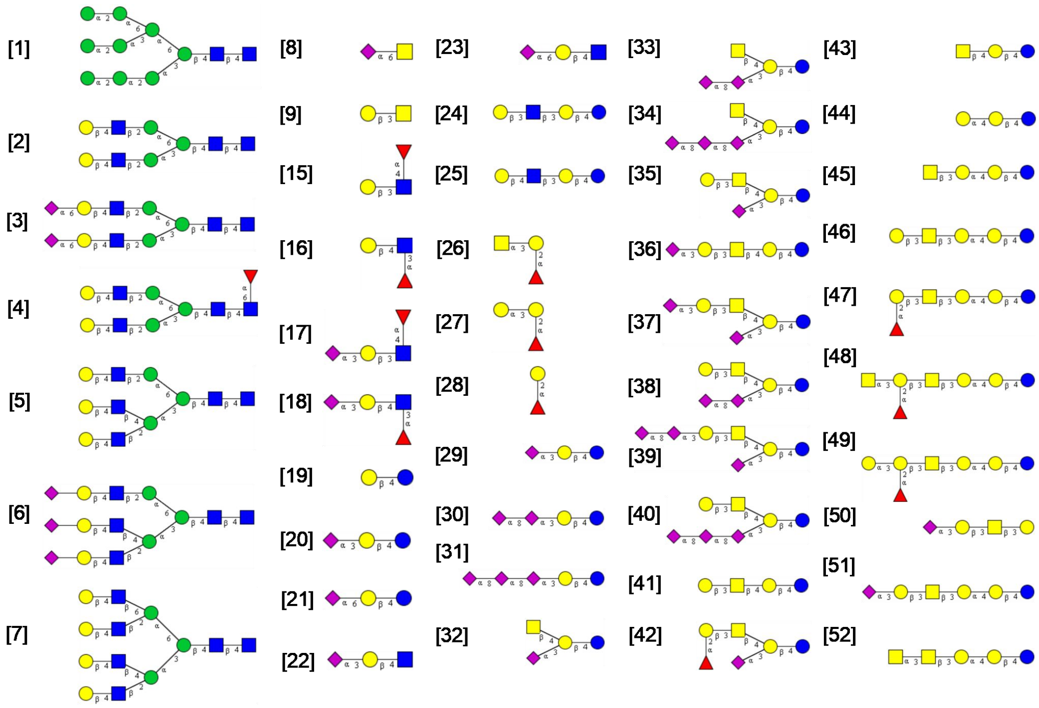

B.

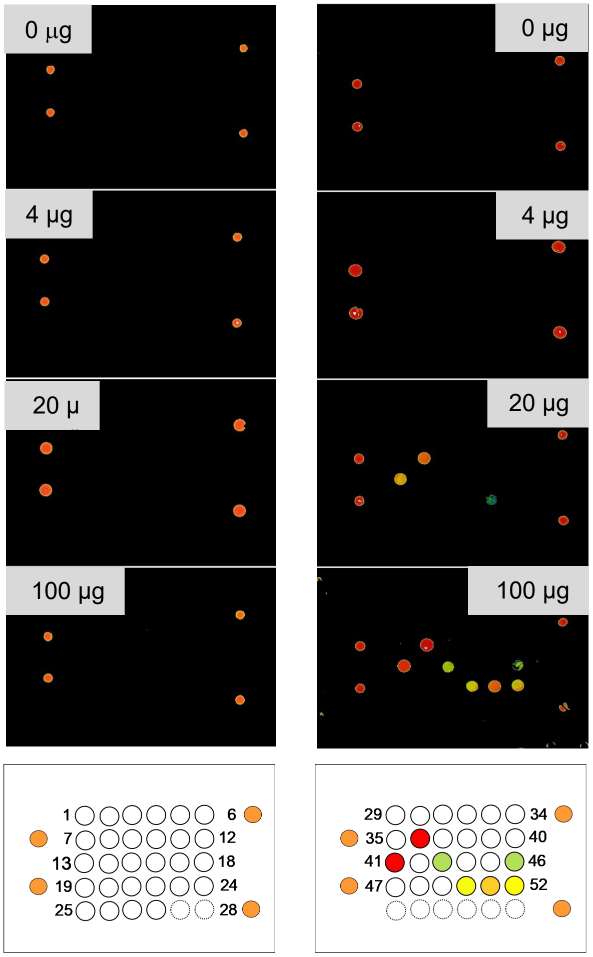

Suppl Fig S3. Glycan-array analysis.
A. List of oligosaccharides for the glycan-array analysis. Circles of green, yellow and navy blue indicate D-Man, D-Gal and D-Glc, respectively. Squares of navy blue and yellow indicate D-GlcNAc and D-GalNAc, respectively. Rhombus (pink) and triangle (red) indicate Neu5Ac and L-Fuc, respectively. The numbering corresponds to Fig 6 and Suppl Fig S3B and Suppl Table 1.

B. Glycan-array analysis. The numbers of each spot on the chip corresponding to the glycans indicated in Suppl Table S1 and Fig 6 in the main text. HiLyte555 Fluoro-labeled SeviL (0-100 μg/ml) applied to glycan tip that is spotted 52 oligosaccharides (the numbering of glycans in A and B corresponds to Fig 6). The binding SeviL to each glycan was detected by an evanescent fluoro scanner, Bio-REX Scan 300. The wavelength of the laser light was selected as same as the Cy3 detection and the exposure time was 300 msec.

Suppl Fig S4

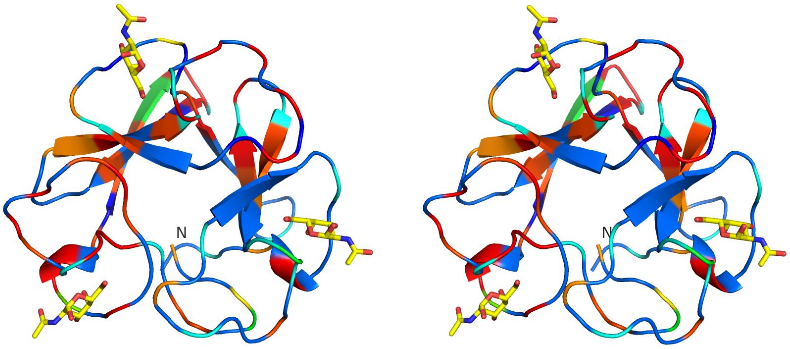

Suppl Fig S4. A homology model of SeviL.
Left: A stereo view of the crystal structure of one subunit of a β-trefoil lectin, MytiLec-1 from *Mytilus galloprovincialis* (PDB 3WMV), with bound ligands (D-GalNAc). **Right**: homology model of SeviL, built with MODELLER (https://salilab.org/modeller/), using MytiLec-1 as a structural template. The model is colored based on the conservation among the sequences of SeviL and its orthologs (see Figure 3). Red indicates 100% conservation and blue indicates very small conservation. The overall primary sequence identity between SeviL and MytiLec was 10%. The sugar-binding sites of MytiLec were not noticeably conserved in the primary structure of SeviL.

Suppl Table S1

Suppl Table S1 List of 52 oligosaccharides used for the glycan-array analysis.

No.	glycan	No.	glycan	No.	glycan	No.	glycan
1	M9	16	Le^x	31	GT3	46	SSEA-3
2	NA2	17	SLe^a	32	GM2	47	Globo-H
3	A2	18	SLe^x	33	GD2	48	Globo-A
4	NA2F	19	Lac	34	GT2	49	Globo-B
5	NA3	20	3SL	35	GM1a	50	SSEA-4 tetraose
6	A3	21	6SL	36	GM1b	51	SSEA-4 hexaose
7	NA4	22	3SLNAc	37	GD1a	52	Forssman antigen
8	STn	23	6SLNAc	38	GD1b		
9	TF	24	LNT	39	GT1a		
10	Heparin	25	LNnT	40	GT1c		
11	De2SHep	26	A	41	asialo-GM1		
12	De6SHep	27	B	42	fucosyl-GM1		
13	DeNSHep	28	H	43	asialo-GM2		
14	DeNS/AcHep	29	GM3	44	Gb3		
15	Le^a	30	GD3	45	Gb4		

Color key:
- N-glycans (1–7)
- O-glycans (8–9)
- glycosaminoglycans (10–14)
- Lewis type oligosaccharides (15–18)
- derivatives of lactose and N-acetyllactosamine (19–25)
- ABH-type oligosaccharides (26–28)
- ganglio-series oligosaccharides (29–43)
- globo-series oligosaccharides (44–52)
